# Validation of the Saint George’s Respiratory Questionnaire in Uganda

**DOI:** 10.1136/bmjresp-2018-000276

**Published:** 2018-07-11

**Authors:** Brooks W Morgan, Matthew R Grigsby, Trishul Siddharthan, Robert Kalyesubula, Robert A Wise, John R Hurst, Bruce Kirenga, William Checkley

**Affiliations:** 1 Division of Pulmonary and Critical Care, School of Medicine, Johns Hopkins University, Baltimore, Maryland, USA; 2 Center for Global Non-Communicable Disease Research and Training, Johns Hopkins University, Baltimore, Maryland, USA; 3 College of Health Sciences, Makerere University, Kampala, Uganda; 4 UCL Respiratory, Division of Medicine, University College London, London, UK; 5 Program in Global Disease Epidemiology and Control, Bloomberg School of Public Health, Johns Hopkins University, Baltimore, Maryland, USA

**Keywords:** COPD epidemiology, respiratory measurement

## Abstract

**Introduction:**

Chronic obstructive pulmonary disease (COPD) will soon be the third leading global cause of death and is increasing rapidly in low/middle-income countries. There is a need for local validation of the Saint George’s Respiratory Questionnaire (SGRQ), which can be used to identify those experiencing lifestyle impairment due to their breathing.

**Methods:**

The SGRQ was professionally translated into Luganda and reviewed by our field staff and a local pulmonologist. Participants included a COPD-confirmed clinic sample and COPD-positive and negative members of the community who were enrolled in the Lung Function in Nakaseke and Kampala (LiNK) Study. SGRQs were assembled from all participants, while demographic and spirometry data were additionally collected from LiNK participants.

**Results:**

In total, 103 questionnaires were included in analysis: 49 with COPD from clinic, 34 community COPD-negative and 20 community COPD-positive. SGRQ score varied by group: 53.5 for clinic, 34.4 for community COPD-positive and 4.1 for community COPD-negative (p<0.001). The cross-validated *c* statistic for SGRQ total score predicting COPD was 0.87 (95% CI 0.75 to 1.00). SGRQ total score was associated with COPD severity (forced expiratory volume in 1 s per cent of predicted), with an *r* coefficient of −0.60 (−0.75, −0.39). SGRQ score was associated with dyspnoea (OR 1.05/point; 1.01, 1.09) and cough (1.07; 1.03, 1.11).

**Conclusion:**

Our Luganda language SGRQ accurately distinguishes between COPD-positive and negative community members in rural Uganda. Scores were correlated with COPD severity and were associated with odds of dyspnoea and cough. We find that it can be successfully used as a respiratory questionnaire for obstructed adults in Uganda.

**Key messages**

- Our Luganda translation of the Saint George’s Respiratory Questionnaire effectively distinguished between those with chronic obstructed pulmonary disease and those without.
- SGRQ total scores were associated with COPD severity and self-reported dyspnoea and cough.

## Background

Chronic obstructive pulmonary disease (COPD) accounted for more than 3 million deaths globally in 2015, 5.3% of the world’s total.^1 2^ As the global burden of COPD increases, special attention must be paid to low/middle-income countries, which are experiencing a unique combination of risk factors: a growing elderly population, urbanisation and increasing tobacco smoking.^3 4^ Additionally, research investigating potential links between biomass fuel use and COPD is ongoing.^5 6^ It has been estimated that the African region experienced the second largest increase in COPD cases between 1990 and 2010 (+102.1%, behind only Eastern Mediterranean among WHO regions).^7^

Within this population, there is a need for a simple tool to identify those who are experiencing lifestyle impairment due to their breathing as part of a plan to diagnose and treat those experiencing chronic respiratory disease. The Saint George’s Respiratory Questionnaire (SGRQ) was designed to evaluate the health impacts of chronic respiratory disease, specifically asthma and COPD.^8^ It is comprised of three sections covering symptoms, physical activities and psychosocial impacts over a set preceding time period and has been shown to correlate to tests of exercise, breathlessness and anxiety/depression.^8^ Thus far, the SGRQ has been validated in over 60 languages. We aimed to translate the 3-month recall version of the SGRQ to Luganda and validate its relationship to airway obstruction in three samples of the Ugandan population: those from the community with no obstruction, those from the community with spirometry-confirmed COPD and those with clinic-confirmed COPD. We further attempted to determine the efficacy of the SGRQ as a screening tool for COPD, indicating those who may require further care.

## Methods

### Study setting

The data for this analysis were collected as part of the Lung Function in Nakaseke and Kampala (LiNK) study, for which the general methods are described elsewhere.^9^ Kampala, the capital of Uganda, has over 400 000 households and a population of around 1.5 million.^10^ Nakaseke is a rural health district with over 40 000 households and nearly 200 000 residents.^10^ It includes a central periurban community and is situated approximately 50 km northwest of Kampala.

### Translation and validation

An initial forward translation of the SGRQ into the Luganda language was obtained from a local translator. This was back-translated and reviewed point by point by the LiNK staff and bilingual field staff, who made edits to ensure that the language was correct and that the spirit and cultural relevance of the questions was retained. The revised copy was reviewed by the local pulmonologist and further minor changes were made.

### Study design

The first language of more than 5.5 million people and spoken by over 6.5 million in Uganda (representing 17% of the 2014 population of 38.5 million), Luganda is the predominant Bantu language in southern and central regions of the country.^11^ As part of the LiNK study, our Luganda SGRQ was administered to participants from the community who tested positive for COPD in the field. For this analysis, fieldworkers were instructed to administer the Luganda SGRQ to a convenience sample of participants from the community who tested negative for COPD. We aimed to collect at least 30 SGRQ tests in each group in order to detect the 16.5-point difference reported in the Nepali SGRQ validation.^12^ Finally, a fieldworker was sent to the lung clinic of Mulago National Referral Hospital to recruit a set of 50 individuals with clinic-confirmed COPD. Inclusion criteria included age ≥30 years and ability to provide informed consent. Exclusion criteria included pregnancy and report of active tuberculosis. Written informed consent was obtained. Spirometry was conducted according to American Thoracic Society/European Respiratory Society guidelines.^13^ All data were collected via Open Data Kit (University of Washington, Seattle, Washington) on tablet computers.

### Definitions

The SGRQ comprises three subsections: the symptoms component covers the effects, frequency and severity of respiratory symptoms; the activity component covers daily activities that cause or are impaired by breathlessness; and the impact component covers social functioning and the psychosocial disturbances associated with their respiratory disease.^14^ The total score, a combination of the three component scores, is presented as a scale of health impairment where 100 represents the worst possible health and 0 represents the best.^14^ All spirometric predicted values are based on the National Health and Nutrition Examination Survey (NHANES) African-American reference population.^15^ COPD was defined as a post-bronchodilator forced expiratory volume in 1 s/forced vital capacity (FEV_1_/FVC) ratio less than the lower limit of normal (bottom 5th percentile of the reference population). Cough was defined as self-report of having cough daily or upon waking in the morning. Dyspnoea was defined as self-report of experience of shortness of breath with physical exertion. Wheeze was defined as self-report of the chest often sounding noisy (wheezy, whistling) when breathing. Phlegm was defined as self-report of frequently coughing up mucus.

### Biostatistical methods

Baseline group differences were obtained via Χ^2^ or t-tests for categorical or continuous variables, respectively. Differences in total and component SGRQ scores between the community COPD-negative, community COPD-positive and clinic COPD-positive groups were obtained via analysis of variance tests. Adjusted differences between the community COPD-positive and negative groups were obtained via linear regression, controlling for age, sex and height. Validity of SGRQ total score and component categories as predictors of COPD status in the community was assessed via unadjusted logistic modelling and receiver operating characteristic (ROC) curves. Based on this outcome, a cut point was chosen and agreement between SGRQ and COPD status using this cut point was established via Fleiss’ kappa test. The association between SGRQ score and COPD severity as defined by per cent predicted FEV_1_ was assessed via Pearson correlation. Analyses were performed in Stata V.13 (StataCorp, College Station, Texas).

## Results

### Participant characteristics

A collection of 49 SGRQs was gathered from the lung clinic of Mulago National Referral Hospital. Additionally, a total of 54 adult participants from our community sample in Nakaseke, Uganda, were included in this analysis for a total sample of 103. Those from the community had a mean age of 49.1 years and were 55.6% female. Those with COPD were older, had a lower body mass index and were more likely to smoke (table 1). There was a 3.48 *z* score difference in FEV_1_/FVC ratio between the COPD-positive and COPD-negative community participants (−3.48 vs 0.00; p<0.001). The only demographic difference between the sample and the parent cohort in Nakaseke was history of post-treatment pulmonary tuberculosis, with a higher prevalence among SGRQ sample participants (online supplementary table 1). The sample did differ in most ways from the parent cohort with respect to lung function, having generally worse scores from prebronchodilator spirometry, which was expected as it contains a much higher prevalence of participants with pulmonary obstruction. Our sample of COPD-positive community participants represents 50% of the 40 identified during the LiNK study in Nakaseke. Those with COPD from the community who were included in our validation sample had slightly more severe obstruction (GOLD criteria) than those who were not included (online supplementary table 2).^16^

10.1136/bmjresp-2018-000276.supp1 Supplementary file 1

**Table 1:** Comparison of demographic factors between community COPD-positive and community COPD-negative participants.

	COPD negative (63.0%, n=34)	COPD positive (37.0%, n=20)	P values
Demographics: % (n) or mean (SD)			
Age (years)	45.7 (8.92)	54.9 (13.6)	0.004
Being female	64.7% (22)	40.0% (8)	0.08
Body mass index (kg/m^2^)	26.4 (5.01)	20.0 (2.99)	<0.001
Primary education or less	76.5% (26)	95.0% (19)	0.08
Household size >3 people	82.4% (28)	50.0% (10)	0.012
Risk factors: % (n)			
Current smoking	2.9 (1)	30.0 (6)	0.004
Obesity (body mass index ≥30 kg/m^2^)	26.5 (9)	0 (0)	0.012
Self-reported HIV	3.5 (1)	14.3 (2)	0.19
Self-reported tuberculosis	3.9 (1)	20.0 (3)	0.09
Household biomass use	97.1 (33)	100 (20)	0.44
Prebronchodilator spirometry: mean (SD)			
FEV_1_ (L)	2.50 (0.47)	1.57 (0.80)	<0.001
% Predicted	0.92 (0.13)	0.62 (0.23)	<0.001
FVC (L)	3.12 (0.50)	2.68 (1.16)	0.06
% Predicted	0.93 (0.11)	0.84 (0.24)	0.05
FEV_1_/FVC ratio	80.1 (6.69)	57.5 (9.98)	<0.001
% Predicted	0.99 (0.08)	0.72 (0.12)	<0.001
Symptoms: % (n)			
Cough	9.1 (3)	35.0 (7)	0.019
Phlegm	10.7 (3)	5.3 (1)	0.51
Wheeze	7.1 (2)	10.5 (2)	0.68
Dyspnoea with exertion	14.3 (4)	26.3 (5)	0.30

P value represents results from t-test or Χ^2^ test.

COPD, chronic obstructive pulmonary disease; FEV_1_, forced expiratory volume in 1 s; FVC, forced vital capacity.

### SGRQ scores between groups

**Figure 1:**
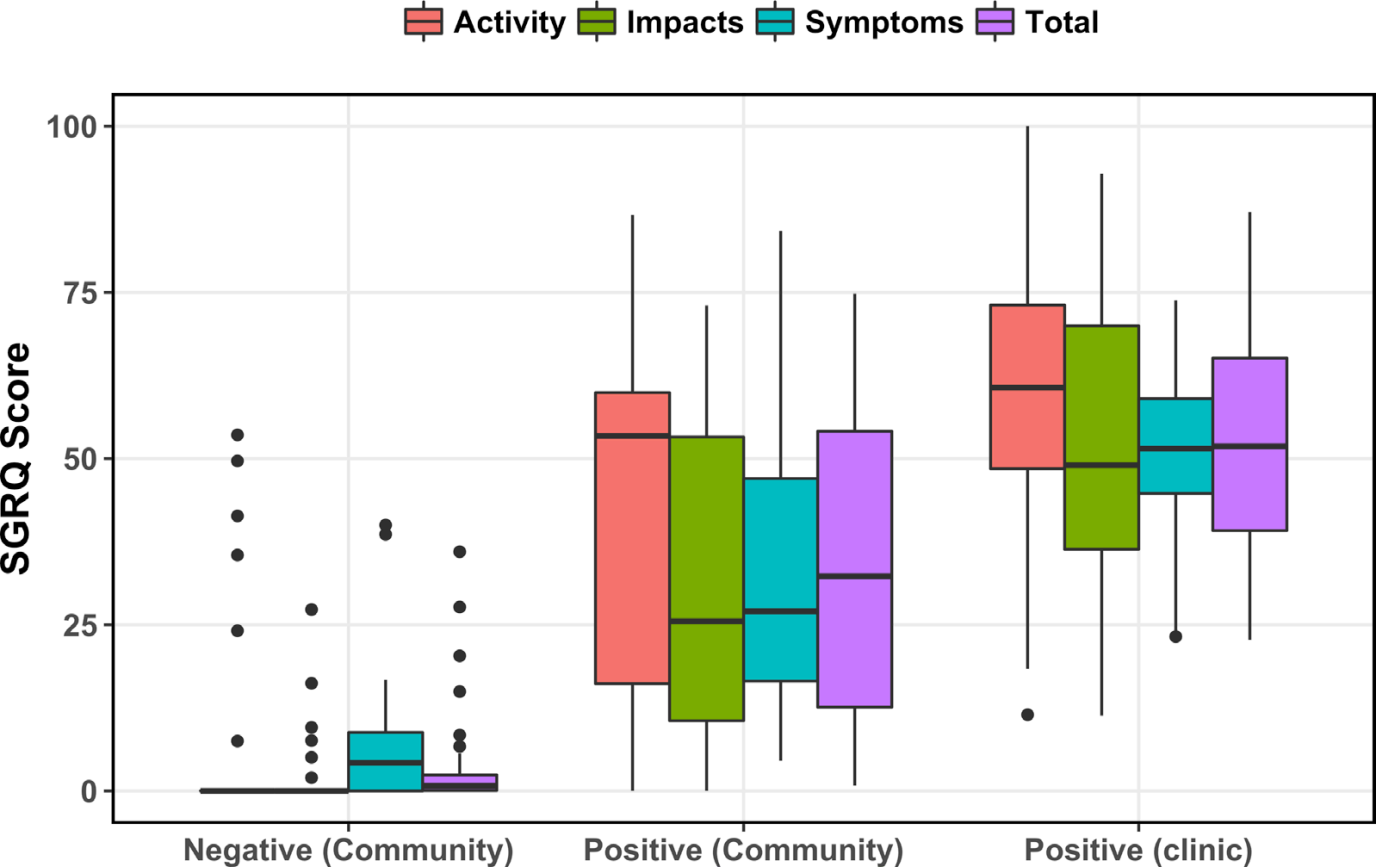
Boxplots of Saint George’s Respiratory Questionnaire (SGRQ) scores between groups. Top and bottom of box represent 75th and 25th percentiles of distribution. Horizontal bar represents median. Dots represent outlying data.

SGRQ total score was highest for the clinic population, followed by the community COPD-positive and community COPD-negative groups (figure 1). The same pattern was seen after breaking total score down into component segments: symptoms score, activity score and impacts score. Significant differences were found between each pairwise comparison of groups, both for total and component scores. In linear regression among community participants, SGRQ scores were higher across the board for those who were COPD positive compared with those who were COPD negative (table 2). Further, SGRQ total score was higher (26.5 points, 95% CI 13.2 to 39.8) among those who smoked daily, adjusted for age, sex and height. SGRQ score was unable to be modelled against biomass fuel smoke exposure due to homogeneity of exposure (97.1% of sample used biomass fuels).

**Table 2:** Differences in SGRQ scores between community COPD-positive and community COPD-negative participants.

	Single variable	Multivariable*
Outcomes: difference in score		
Symptoms	27.5 (16.9–34.7)	29.3 (17.3–36.9)
Activity	36.8 (23.6–47.4)	37.2 (22.7–47.8)
Impacts	30.0 (19.9–37.5)	29.2 (18.1–37.4)
Total	30.3 (21.3–39.2)	29.9 (20.2–39.6)

P value represents results from regression analyses.

*Multivariable models adjusted for age, sex and height.

COPD, chronic obstructive pulmonary disease; SGRQ, Saint George’s Respiratory Questionnaire.

**Figure 2:**
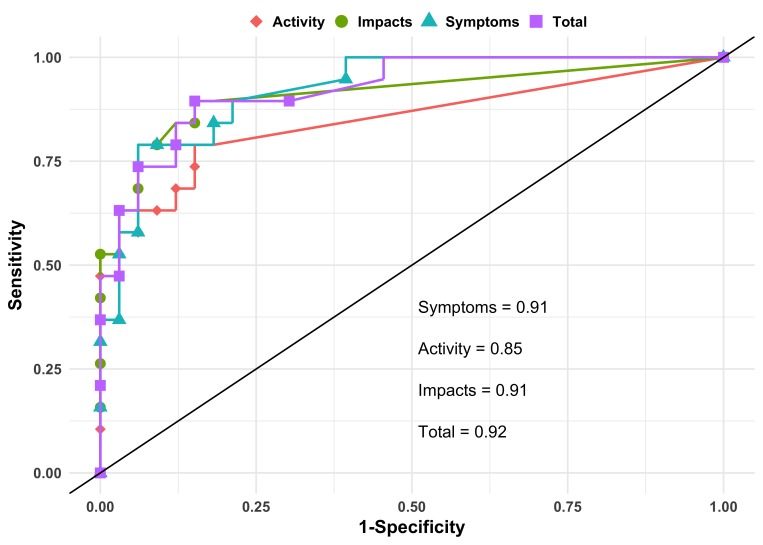
Receiver operating characteristic (ROC) curves for Saint George’s Respiratory Questionnaire (SGRQ) category scores predicting chronic obstructive pulmonary disease (COPD). Points represent combination of sensitivity and specificity of prediction of COPD at each value of SGRQ total and component score in analysis.

From single variable logistic regression and ROC analysis, the *c* statistic for total SGRQ score as a predictor of COPD status was 0.92 (95% CI 0.85 to 0.99) (figure 2). The *c* statistic for component SGRQ scores alone was 0.91 (95% CI 0.83 to 0.98) for symptoms, 0.91 (95% CI 0.82 to 0.99) for impacts and 0.85 (95% CI 0.74 to 0.96) for activities. Sensitivity analysis with 10-fold cross-validation revealed slightly lower though still strong *c* statistics: 0.87 (95% CI 0.75 to 1.00) for SGRQ total score with component scores ranging from 0.77 to 0.90. The optimal cut point for predicting COPD based on this sample was 6.2 for both the Liu and Youden methods, which, when applied to the source sample, lead to a Fleiss’ kappa score for classification agreement of 0.72 (95% CI 0.45 to 0.99).

### SGRQ and lung function

Total SGRQ score was linearly associated with COPD severity among all community participants, with a Pearson’s correlation coefficient of −0.60 (95% CI −0.75 to −0.39) (online supplementary figure 1). After stratifying by group, no association was seen between SGRQ score and lung dysfunction among COPD-negative participants (*r*=0.09; 95% CI −0.27 to 0.42) while a negative correlation was retained among COPD-positive participants (*r*=−0.38; 95% CI −0.71 to 0.09).

10.1136/bmjresp-2018-000276.supp2 Supplementary file 2

### SGRQ and symptoms

**Figure 3:**
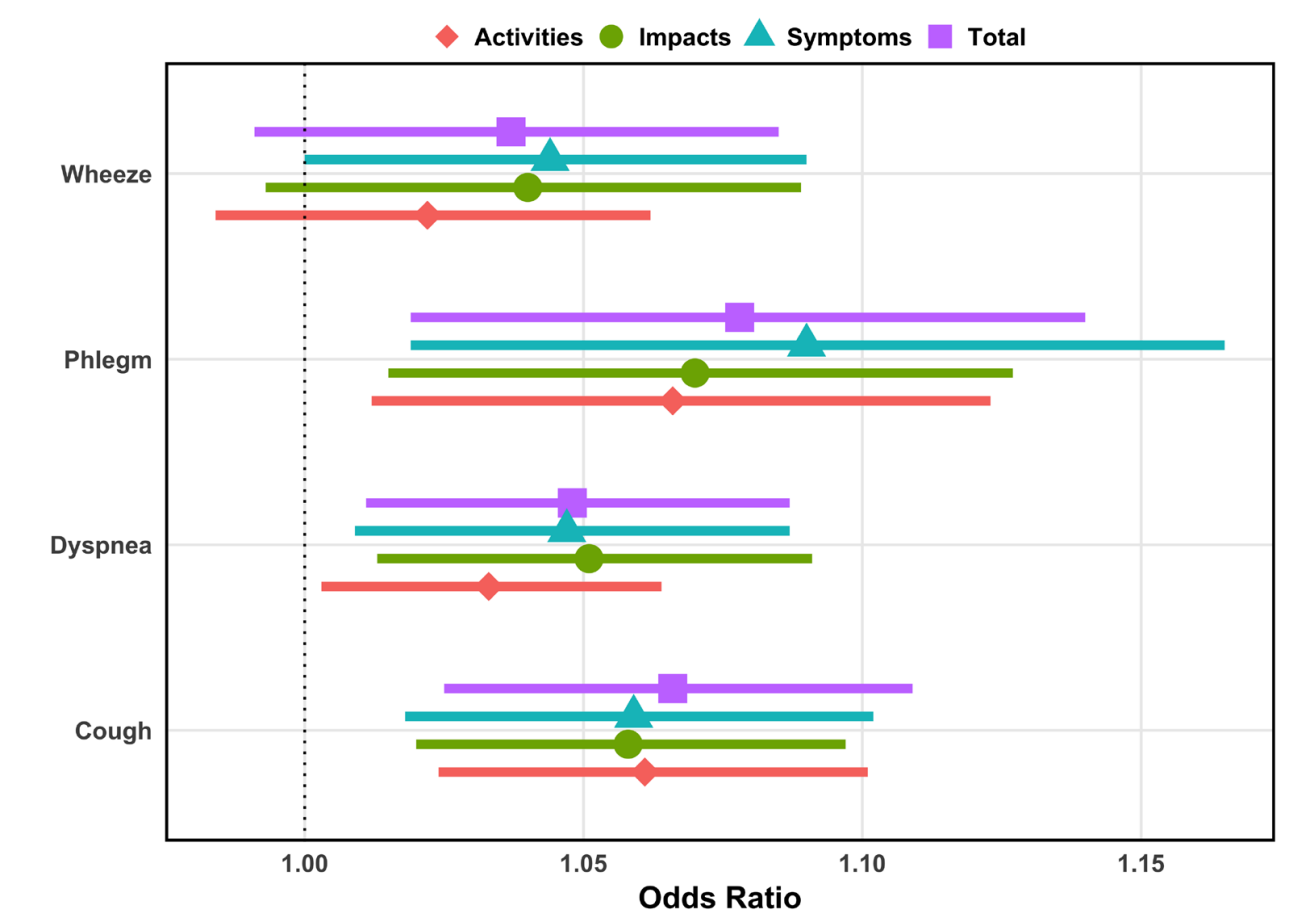
Association of Saint George’s Respiratory Questionnaire (SGRQ) total and category scores with respiratory symptoms. The diamonds represent the increase in odds for having the respiratory symptom outcome based on a unit increase in SGRQ score from multivariable logistic regression analysis. The coloured bars represent the 95% CI of the estimate. All models are adjusted for age and sex.

Adjusted by age and sex, SGRQ total score was a significant linear predictor of dyspnoea (OR 1.05/point; 95% CI 1.01 to 1.09), cough (OR 1.07; 95% CI 1.03 to 1.11) and phlegm (OR 1.08/point, 95% CI 1.02 to 1.14) and trended towards an association with wheeze (OR 1.04; 95% CI 0.99 to 1.09) (figure 3 and online supplementary table 3). Within the component sections, symptom scores were positively associated with each respiratory outcome while activity and impact scores were associated with phlegm, cough, and dyspnoea alone.

## Discussion

We developed and validated the SGRQ in Luganda. Significant differences were found in total and component scores between COPD-positive and negative population-sampled groups and we observe an ability to accurately distinguish between the two in our sample. Further, we saw a correlation between increasing SGRQ scores and decreasing FEV_1_ per cent of predicted. We anticipate that it can be successfully used as a respiratory questionnaire for obstructed adults in Uganda.

Data on COPD in Uganda are limited to two studies: the LiNK study and the FRESH AIR study in Masindi.^9 17^ Prevalence estimates for COPD range from 6% to 16%, yet the Clinical COPD Questionnaire—used in FRESH AIR—is the only validated measure assessing symptoms and quality of life in these areas.^17^ Our translation of the SGRQ was effective in discriminating between COPD-positive and negative participants in our sample from Nakaseke, Uganda. The *c* statistic of 0.92 for the SGRQ total score is high; however, it is expected to be high when applied to the same sample from which it was calculated. In sensitivity analyses using 10-fold cross-validation, the area under the curve remained high but was a more reasonable 0.88. The cross-validated score remained much higher than the 0.77 found by Sherpa *et al* and the 0.69 found by Weatherall *et al*.^12 18^ One potential contributing factor is that those studies used a fixed cut-off of 70% for FEV_1_/FVC ratio to define COPD whereas we chose to use the lower limit of normal based on the NHANES African-American reference population.^15^

Our finding of a strong correlation coefficient of −0.60 between SGRQ total score and FEV_1_ per cent of predicted for all participants mimics the results of other studies and indicates an important relationship between SGRQ score and severity of airway obstruction.^8 18–21^ Within this, the −0.38 correlation coefficient we found among COPD-positive community members falls within the range of −0.27 to −0.45 previously published.^21–24^

Although the method of modelling differed, our finding of a relationship between a higher SGRQ total score and OR of reporting dyspnoea with exertion agrees with previous studies reporting significant correlations between Saint George’s scores and measures of breathlessness.^8 20 24–27^ Similarly, in this analysis, the SGRQ total score and all component scores were positively associated with cough, as found in the original SGRQ study by Jones and colleagues.^8^ In our sample, only symptom component scores were associated with wheezing, though previous studies had found relationships with total score and other components as well.^8 26^

Our study has several strengths. Nesting within the LiNK study ensured high-quality data (through quality control measures) and standardised administration of spirometry. The inclusion of both clinically validated COPD and community COPD-negative groups in addition to our community COPD-positive group allowed us to look at between-group trends in scoring, unlike validation studies which only included those with COPD.^19–21 23 28 29^ We were also capable of linking the SGRQ to reported symptoms, which was not often included in other validation studies. This analysis had some shortcomings as well. We did not collect demographic or lung function information on clinic participants (who were not enrolled in the LiNK study) so we were unable to include them in analyses concerning regressions or correlations. We also did not hit our initial target sample of COPD-positive community members before LiNK study recruitment ended due to restarting data collection secondary to scoring issues with our original version of the form. Even so, we were able to detect large differences between COPD-positive and negative groups as well as see a linear relationship between SGRQ score and breathing restriction severity. Finally, we were limited to cross-sectional data. This was sufficient for drawing the correlations we have laid out in this paper, but individual follow-up data would allow for a more detailed investigation into how SGRQ scores relate to changing lung function over time, including exacerbations.^20 21 23 28 29^

In summary, the Luganda translation of the SGRQ correlates well with airway obstruction and report of respiratory symptoms and can be an effective respiratory questionnaire for use in Uganda.
