# Supplementary material for: Validation of the Saint George’s Respiratory Questionnaire in Uganda
Source: BMJ Open Respir Res. 2018 Jul 11;5(1):e000276. doi: 10.1136/bmjresp-2018-000276 (PMC6045769; doi:10.1136/bmjresp-2018-000276)
Supplement: Supplementary file 1 [file bmjresp-2018-000276supp001.pdf]

Supplementary Table 1. Comparison of demographic factors between sampled community members and LiNK cohort participants from Nakaseke, Uganda. *P* value represents results from t-test or chi-squared test.

	SGRQ Sample (5.8%, n=54)	Balance of Cohort (94.2%, n=877)	*p* value
**Demographics:** *% (n) or mean (sd)*			
Age (years)	49.1 (11.6)	49.2 (11.4)	0.96
Being Female	55.6% (30)	54.7% (480)	0.91
Body mass index (kg/m^2)	24.0 (5.4)	23.9 (4.6)	0.94
Primary Education or Less	83.3% (45)	79.6% (697)	0.50
Household Size >3 People	70.4% (38)	67.1% (588)	0.62
**Risk Factors:** *% (n)*			
Current Smoking	13.0% (7)	15.2% (133)	0.66
Obesity (Body mass index ≥30 kg/m^2)	16.7% (9)	10.6% (93)	0.17
Self-Reported HIV	7.0% (3)	10.5% (74)	0.46
Self-Reported Tuberculosis	9.8% (4)	2.4% (18)	0.005
Household Biomass Use	98.2% (53)	99.6% (872)	0.10
**Pre-Bronchodilator Spirometry:** *mean (sd)*			
FEV1 (L)	2.16 (0.75)	2.44 (0.65)	0.003
% Predicted	0.81 (0.23)	0.94 (0.19)	<0.001
FVC (L)	2.95 (0.83)	3.11 (0.77)	0.14
% Predicted	0.90 (0.17)	0.96 (0.17)	0.007
FEV1/FVC Ratio	71.7 (13.6)	78.2 (7.98)	<0.001
% Predicted	0.89 (0.16)	0.97 (0.09)	<0.001
**Symptoms:** *% (n)*			
Cough	18.9% (10)	6.8% (52)	0.001
Phlegm	9.4% (5)	3.9% (29)	0.12
Wheeze	8.5% (4)	2.9% (22)	0.037
Dyspnea with exertion	19.2% (9)	8.8% (66)	0.018

Supplementary Table 2. Comparison of COPD severity[a] between sampled COPD-positive participants and all COPD-positive participants from Nakaseke, Uganda. *P* value represents result from chi-squared test.

	In Sample (22.4%, n=19)[b]	Out of Sample (77.7%, n=66)	*p* value
**COPD Severity:** *% (n)*			0.01
Mild	21.1% (4)	56.1% (37)	
Moderate	52.6% (10)	36.4% (24)	
Severe	26.3% (5)	7.6% (5)	
Very Severe	0% (0)	0% (0)	

[a] **Using GOLD criteria for post-bronchodilator spirometry**
[b] **One dropped for poor-quality spirometry**

Supplementary Table 3. Association of SGRQ total and category scores with respiratory symptoms. The results represent the increase in odds for having the respiratory symptom outcome based on a unit increase in SGRQ score from multivariable logistic regression analysis. All models are adjusted for age and sex.

	Wheeze 8.5% (4)	Phlegm 9.4% (5)	Dyspnea 19.2% (9)	Cough 18.9% (10)
**Respiratory Symptoms:** *odds ratio (95% CI)*				
Symptoms	1.04 (1.00 – 1.09)	1.09 (1.02 – 1.17)	1.05 (1.01 – 1.09)	1.06 (1.02 – 1.10)
Activity	1.02 (0.98 – 1.06)	1.07 (1.01 – 1.12)	1.03 (1.00 – 1.06)	1.06 (1.02 – 1.10)
Impacts	1.04 (0.99 – 1.09)	1.07 (1.02 – 1.13)	1.05 (1.01 – 1.09)	1.06 (1.02 – 1.10)
Total	1.04 (0.99 – 1.09)	1.08 (1.02 – 1.14)	1.05 (1.01 – 1.09)	1.07 (1.03 – 1.11)
