# Supplementary figures and images for: Validation of the Saint George’s Respiratory Questionnaire in Uganda
Source: BMJ Open Respir Res. 2018 Jul 11;5(1):e000276. doi: 10.1136/bmjresp-2018-000276 (PMC6045769; doi:10.1136/bmjresp-2018-000276)

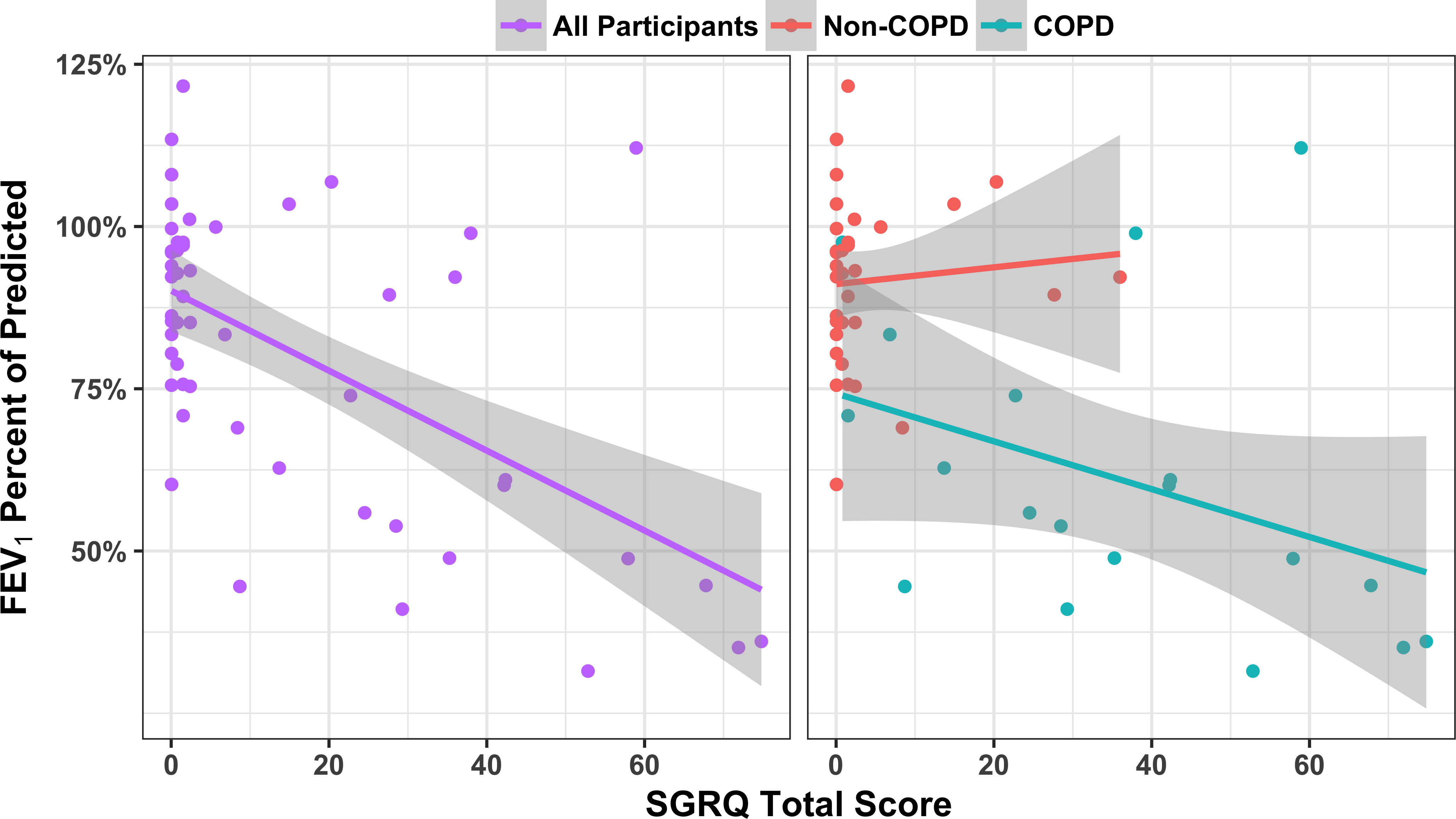

Supplement: Supplementary file 2 [file bmjresp-2018-000276supp002.jpg]
